# Integrated Network Toxicology and Transcriptomics Reveal Molecular Mechanisms of Cadmium-Exposed Liver Injury in Swine

**DOI:** 10.3390/ani16030414

**Published:** 2026-01-28

**Authors:** Nan Wang, Xuehan Jiang, Xiaoxiao Chen, Biner Zhao, Jingzeng Cai, Ziwei Zhang

**Affiliations:** 1 College of Veterinary Medicine, Northeast Agricultural University, Harbin 150030, China; letong2001cn@163.com (N.W.); xuehan415@163.com (X.J.); cc13337816646@163.com (X.C.); 18845789909@163.com (B.Z.); 2 Key Laboratory of the Provincial Education, Department of Heilongjiang for Common Animal Disease Prevention and Treatment, Harbin 150038, China

**Keywords:** cadmium, hepatotoxicity, network toxicology, transcriptome sequencing, swine

## Abstract

Cadmium (Cd) is an environmental toxicant that poses significant risks to food safety and public health through its bioaccumulation in the food chain. The liver is a primary target for chronic Cd toxicity, yet the system-level mechanisms, particularly in physiologically relevant swine models, remain incompletely understood. This study employed an integrated multi-omics approach to elucidate the mechanisms of Cd-exposed hepatotoxicity in weaned piglets. We combined histopathological examination, transmission electron microscopy, and transcriptome sequencing. Our results revealed severe hepatic damage, characterized by disorganized architecture, vacuolar degeneration, mitochondrial dysfunction, and autophagic activation. Network toxicology predicted 3727 potential targets of Cd-exposed liver injury, while transcriptomics identified 1092 differentially expressed genes (DEGs). Crucially, the convergent analysis of both datasets demonstrated that the PI3K-Akt signaling pathway was the central hub, pinpointing it as a pivotal mechanism in Cd-driven hepatotoxicity. Functional enrichment analyses further highlighted dysregulation in immune-inflammatory responses, lipid metabolism, and oxidative stress. Our findings provide a comprehensive systems-level perspective on chronic Cd hepatotoxicity in a translational swine model. We propose the PI3K-Akt pathway and other identified core targets (EGFR, histones, ribosomal proteins) as critical biomarkers for monitoring Cd contamination in swine production chains, offering valuable insights for environmental risk assessment and agricultural product safety.

## Simple Summary

Cadmium is a toxic metal that can enter livestock through contaminated soil, water, or feed, raising food-safety concerns. We aimed to explain how long-term cadmium exposure damages pig liver and to find early warning signals for monitoring. We fed piglets a cadmium-containing diet for 40 days and evaluated liver injury using histopathology, ultrastructural imaging, and transcriptomic profiling. Cadmium exposure disrupted normal liver structure, caused swollen and fatty liver cells, damaged the cell’s energy-producing structures, and increased the cell’s recycling process. The blood marker of liver injury increased, and more than one thousand genes changed their activity. By combining gene results with computer analysis, we identified a central control system that coordinates cell survival, energy use, and stress responses as a key driver of cadmium-related liver injury. These results help explain why cadmium-contaminated environments can threaten the safety of animal products and provide practical molecular markers that may improve monitoring and prevention in pig production chains.

## 1. Introduction

Cadmium (Cd), a pervasive environmental toxicant originating from industrial emissions and contaminated soil, bioaccumulates in the food chain and poses significant threats to human and animal health [1]. Due to its high residence time and low clearance rate across organs, Cd exhibits a biological half-life of 10–30 years, driving chronic toxicity [2]. The liver and kidneys are primary targets of Cd exposure, exhibiting acute and chronic sensitivity through mechanisms involving oxidative stress, DNA damage, endoplasmic reticulum stress, and dysregulated autophagy [3,4,5]. Beyond liver and kidney injury, Cd exposure impairs the reproductive system, causing Sertoli cell damage and testosterone reduction in males and follicular atresia in females [6,7,8].

While commercial swine production operates under controlled conditions, cadmium feed contamination remains a documented food safety concern. Previous studies have reported detectable Cd levels in pig livers from standard production systems, with some samples exceeding EU maximum residue limits [9]. Furthermore, dietary formulations containing contaminated feed ingredients, such as certain rye varieties, have been shown to significantly increase Cd accumulation in porcine liver and kidney tissues [10]. The maximum permitted Cd level in compound feed is 1 mg/kg; however, contamination via phosphate supplements and polluted feedstocks creates realistic exposure scenarios.

Accounting for roughly 15% of global animal liver consumption [9,11], pig liver, whose safety and quality resonate far beyond the farm gate, makes the threat of Cd contamination a public-health concern of broad significance. From a food-safety viewpoint, Cd preferentially accumulates in the liver, an edible offal with substantial human consumption, making hepatic injury in pigs directly relevant to the safety of animal-derived foods. As the first-pass metabolic organ, the liver is not only an early site for Cd accumulation but also a primary target for its chronic toxicity. In the liver, Cd induces mitochondrial dysfunction and disrupts lipid metabolism, amplifying structural and functional damage [12,13]. However, current studies focus predominantly on isolated biochemical markers, lacking molecular analysis at the systems level [10]. This gap is exacerbated by the limited application of advanced toxicogenomic approaches in livestock species. Swine models, sharing >80% genomic homology and analogous hepatic metabolism with humans, offer superior translational relevance over rodents [14]. However, no study has comprehensively integrated histopathology, subcellular dynamics, and transcriptome-network interactions to decode Cd-driven hepatotoxicity in pigs.

Notably, our exposure paradigm is intended as a hazard-oriented mechanistic model to enable pathway resolution and biomarker discovery for food-safety surveillance, with direct applicability to Cd contamination events documented in commercial swine production systems. Accordingly, we hypothesized that sub-chronic dietary Cd exposure induces porcine liver injury by disrupting mitochondrial homeostasis and immune–metabolic signaling, with the PI3K–Akt pathway acting as a central regulatory hub. To test this hypothesis and address the above knowledge gap, we integrated histopathology, transmission electron microscopy (TEM), network toxicology, and RNA sequencing to delineate Cd-associated liver injury in weaned piglets. Our study provides a multi-layer atlas of Cd hepatotoxicity in swine, revealing evolutionarily conserved damage pathways and identifying potential biomarkers for monitoring Cd contamination in swine production chains.

## 2. Materials and Methods

### 2.1. Animals and Treatments

Ten 6-week-old male weaned piglets were obtained from the Laboratory Animal Center (Harbin Veterinary Research Institute) and balanced for body weight, age, parity, and genetic background. Animals were maintained at 20 ± 2 °C under a 12 h light/dark cycle with ad libitum access to a standard basal diet and water. After 7 days of acclimation, piglets were randomly assigned to a control group (CON) or a cadmium group (CD; *n* = 5 per group). The CD group received a basal diet supplemented with CdCl_2_ (20 mg/kg; purity >99%; Shanghai Chemical Reagent Company, Shanghai, China) for 40 days, following our established porcine exposure regimen [15,16]. On day 40, livers were collected; tissue aliquots were fixed in 10% buffered neutral formalin (A-CSH466, Shanghai Chemical Reagent Company, Shanghai, China) for histology and immunofluorescence, while the remaining samples were stored at −80 °C for subsequent analyses. All procedures were approved by the Institutional Animal Care and Use Committee of Northeast Agricultural University (SRM-11-NEAUEC20020329).

### 2.2. Histopathological Examination (H&E)

Formalin-fixed liver tissues were paraffin-embedded and cut into 5 µm sections, followed by deparaffinization/rehydration and H&E staining. Sections were examined under light microscopy, and images were recorded using Case Viewer (version 4.3; 3DHistech Ltd., Budapest, Hungary).

### 2.3. Ultrastructural Examination

Small liver blocks (~1 mm^3^) were fixed in 2.5% glutaraldehyde (Electron Microscopy Sciences, Hatfield, PA, USA) in phosphate buffer (4 °C), post-fixed with 1% osmium tetroxide (Electron Microscopy Sciences, Hatfield, PA, USA), and contrasted with 4.8% uranyl acetate (Electron Microscopy Sciences, Hatfield, PA, USA). Ultrathin sections (50–70 nm) were prepared, mounted, and post-stained with lead citrate (Electron Microscopy Sciences, Hatfield, PA, USA) before ultrastructural examination by a transmission electron microscope (model HT7800; Hitachi High-Tech Corporation, Tokyo, Japan).

### 2.4. Network Toxicology

The SMILES string of cadmium (Cd) was downloaded from the PubChem database (National Center for Biotechnology Information, National Library of Medicine, Bethesda, MD, USA) and used to retrieve putative Cd-associated targets from the Comparative Toxicogenomics Database (CTD; http://ctdbase.org/, accessed on 22 December 2025; MDI Biological Laboratory, Salisbury Cove, ME, USA), SwissTargetPrediction (http://www.swisstargetprediction.ch/, accessed on 22 December 2025; Swiss Institute of Bioinformatics, Lausanne, Switzerland), and the STITCH database (http://stitch.embl.de/, accessed on 22 December 2025; EMBL, Heidelberg, Germany), followed by deduplication. Liver injury-related genes were collected from the GeneCards database (https://www.genecards.org/, accessed on 22 December 2025; Weizmann Institute of Science, Rehovot, Israel) using the terms “liver damage”, “liver toxicity”, and “liver injury”; duplicated entries were removed, and genes with relevance scores above the median were retained. Targets with relevance scores above the median were considered highly associated with hepatotoxicity.

Candidate targets were subjected to GO enrichment analysis using the Gene Ontology Consortium resource (http://geneontology.org/, accessed on 22 December 2025; Gene Ontology Consortium) [17] and KEGG pathway analysis using KOBAS (version 3.0; Peking University, Beijing, China) [18]. Enrichment results with adjusted *p* values (Padj) < 0.05 were considered significant.

### 2.5. RNA Extraction, Library Construction, and Illumina Sequencing

Total RNA was isolated from liver tissues using TRIzol (15596-026, Invitrogen, Waltham, MA, USA). RNA concentration/purity and integrity were assessed using a NanoDrop 2000 (Thermo Fisher Scientific, Waltham, MA, USA) and an Agilent 2100 Bioanalyzer RNA Nano 6000 kit (Agilent Technologies, Santa Clara, CA, USA). Libraries were quantified by Qubit 2.0, diluted to 1 ng/µL, checked for insert size on the Bioanalyzer, and further quantified by qPCR (library activity > 2 nM). Indexed libraries were clustered on a cBot system (TruSeq PE Cluster Kit v3-cBot-HS, Illumina, Inc., San Diego, CA, USA) and sequenced as 150 bp paired-end reads on an Illumina platform (Illumina, Inc, CA, USA). Raw FASTQ reads were processed by the sequencing provider (TIANGEN, Beijing, China). Adapter sequences and low-quality bases were removed using Trimmomatic, and basic quality metrics (Q20, Q30, and GC content) were summarized on the resulting clean reads. Clean reads were aligned to the reference genome using Hisat2 (version 2.0.5), gene-level counts were generated with HTSeq-count (version 0.13.5), and expression was reported as FPKM.

### 2.6. Differential Expression mRNAs Screening and Enrichment Analysis

Expression values were summarized as FPKM. Differential expression between CON and CD was assessed using the DESeq2 package (version 1.34.0; Bioconductor, Fred Hutchinson Cancer Research Center, Seattle, WA, USA) [19], with Benjamini–Hochberg correction applied to control the false discovery rate; genes with Padj < 0.05 were defined as differentially expressed. A heatmap was generated using z-score-transformed FPKM values of the significant DEGs. In addition, read counts were normalized using edgeR scaling factors, and differential expression was also tested with edgeR (version 3.18.1; Bioconductor) using Benjamini–Hochberg adjustment. Genes meeting Padj < 0.05 and an absolute fold change ≥ 2 were considered significant. Volcano plots were produced using OriginPro software (version 2018; OriginLab Corporation, Northampton, MA, USA). Functional enrichment (GO and KEGG) of DEGs was performed as described in Section 2.4.

### 2.7. Protein Interaction Network Analysis

Differentially expressed genes were submitted to the STRING database (version 11.5; https://string-db.org/, accessed on 22 December 2025; ELIXIR, Cambridge, UK) to construct the PPI network, which was then visualized in Cytoscape software (version 3.10.1; Cytoscape Consortium, San Diego, CA, USA). Hub nodes were defined as those with degree, betweenness centrality, and closeness centrality above the network median, and key modules were extracted using the MCODE plugin (Cytoscape App Store; Bader Lab, University of Toronto, Toronto, ON, Canada) [20].

### 2.8. Quantitative RT-PCR (qRT-PCR) Analysis

Total RNA from liver tissues was extracted with TRIzol (Thermo Fisher Scientific, Waltham, MA, USA) and reverse-transcribed to cDNA using the TaKaRa Reverse Transcription Kit (RR036A; Takara Bio Inc., Shiga, Japan). qPCR was performed on a LineGene 9600 Plus system (FQD-96A; Bioer Technology Co., Ltd., Hangzhou, China) using Talent qPCR PreMix (SYBR Green) (A25742, TIANGEN, Beijing, China). Relative expression was calculated by the 2^–∆∆Ct^ method. Each assay was conducted in triplicate, and primer sequences are provided in Table A1.

### 2.9. Detection of Serum Biochemical Indexes

Whole blood was kept at 4 °C for 1 h and centrifuged at 3000 rpm for 15 min to obtain serum. Serum AST and ALT were measured (100 µL per assay) using a biochemical analyzer (model AU680; Beckman Coulter, Inc., Brea, CA, USA).

### 2.10. Statistical Analysis

All experimental data are presented as mean ± standard deviation. Statistical analyses were performed using GraphPad Prism software (version 9.0; GraphPad Software, San Diego, CA, USA) and R software (version 4.2.0; R Foundation for Statistical Computing, Vienna, Austria). Differences were evaluated by unpaired Student’s *t*-test for parametric data or Mann–Whitney U test for non-parametric data, with statistical significance set at *p* < 0.05. For transcriptomic data, differential gene expression was analyzed using the DESeq2 package (version 1.34.0; Bioconductor, Fred Hutchinson Cancer Research Center, Seattle, WA, USA) with Benjamini–Hochberg multiple testing correction. Functional modules were extracted using the MCODE plugin (Cytoscape App Store; Bader Lab, University of Toronto, Toronto, ON, Canada) with default parameters. For qRT-PCR validation, relative gene expression and correlation with RNA-seq data were assessed by Pearson’s correlation coefficient. All experiments included at least 3 biological replicates.

## 3. Results

### 3.1. Cd-Exposed Histopathological Alterations in Liver

**Figure 1:**
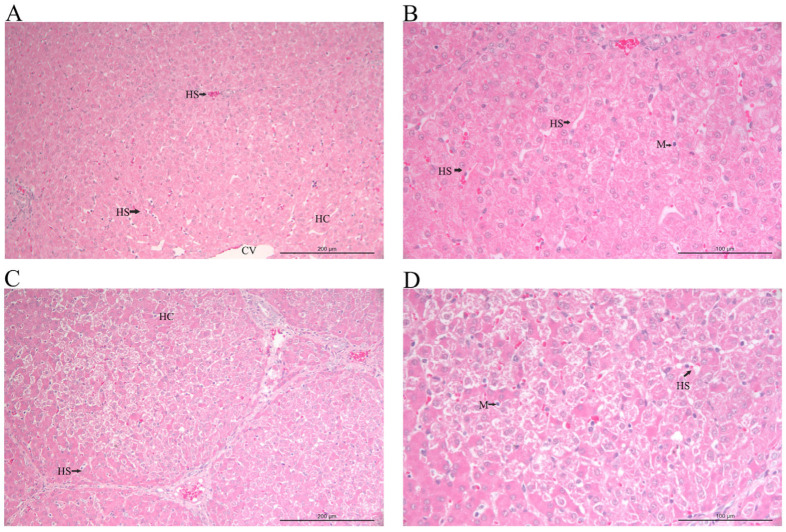
Cd-exposed histopathological changes in swine liver tissues: (**A**–**D**) representative H&E staining of liver sections from CON (**A**,**B**) and CD (**C**,**D**). Macrophage (M); central vein (CV); hepatic cords (HC); hepatic sinusoid (HS).

Compared to the control group, the Cd-exposed group exhibited significant pathological alterations in liver tissue (Figure 1A–D). Low-magnification microscopy revealed disorganized hepatic lobule architecture, disorganized arrangement of hepatic plates, and areas characterized by hepatocyte swelling, vacuolar degeneration, and scattered necrotic foci. Mild to moderate inflammatory cell infiltration accompanied these changes in the portal areas. High-magnification examination demonstrated numerous small lipid droplets within the cytoplasm of hepatocytes. Some nuclei displayed condensation and fragmentation (pyknosis and karyorrhexis), along with mild sinusoidal congestion. In contrast, control group hepatocytes maintained normal morphology, with hepatic cords arranged in a regular radial pattern, and showed no evidence of degeneration, necrosis, or inflammatory infiltration.

### 3.2. Ultrastructural Changes in Cd-Exposed Hepatocytes

**Figure 2:**
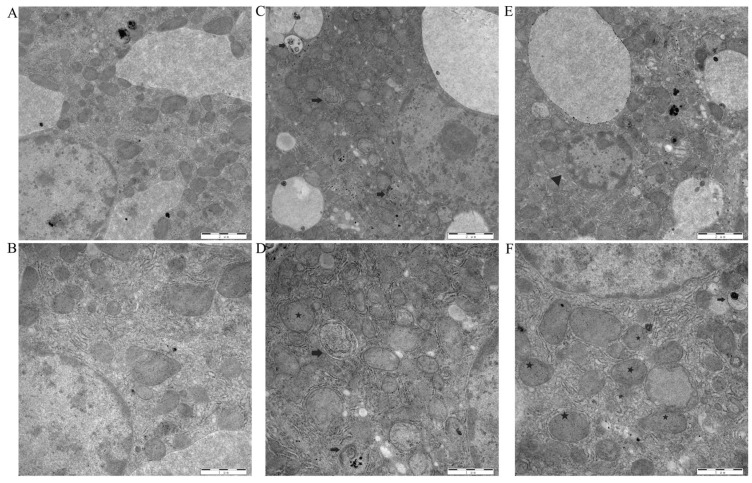
Cd-exposed ultrastructural changes in swine liver tissues; TEM images of hepatocytes from CON (**A**,**B**) and CD (**C**–**F**) liver tissues. Nuclear Shrinkage (△); mitochondrion (★); autophagic vesicles (→).

TEM revealed distinct ultrastructural differences between the groups. Hepatocytes in the control group exhibited regular nuclear morphology with evenly dispersed chromatin, intact mitochondria displaying dense matrices and well-defined cristae, and cytoplasm containing only occasional normal lysosomes without observable autophagosomes (Figure 2A,B). In contrast, the Cd-exposed group displayed marked subcellular toxicity that pronounced nuclear pyknosis with chromatin highly condensed and marginated beneath the nuclear membrane; widespread mitochondrial swelling accompanied by matrix vacuolization and fragmentation or dissolution of cristae, with some organelles exhibiting spherical transformation; and cytoplasm containing numerous autophagosomes/autolysosomes enclosing damaged mitochondria and membrane debris, indicating enhanced autophagic activity (Figure 2C–F). These observations demonstrate that toxin-induced mitochondrial damage and consequent autophagy activation represent key subcellular events in hepatocellular injury.

### 3.3. The Effect of Cd Exposure on Hepatic Injury Serum Markers

**Figure 3:**
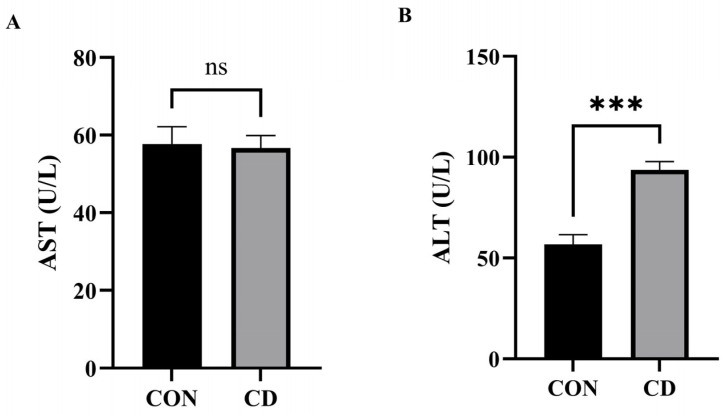
Serum levels of hepatic injury markers: (**A**) AST; (**B**) ALT. *** Represents *p* < 0.001; ns Represents not significant (*p* > 0.05).

As shown in Figure 3, Cd exposure resulted in a differential impact on serum liver enzyme activities. While no statistically significant alteration in AST activity was observed in the CD compared to CON (Figure 3A), the activity of ALT was markedly elevated (*p* < 0.001) (Figure 3B). This distinct elevation in ALT, a more specific indicator of hepatocellular damage, points to Cd-exposed hepatocyte injury.

### 3.4. Network Toxicology Analysis of Cd Hepatotoxicity

**Figure 4:**
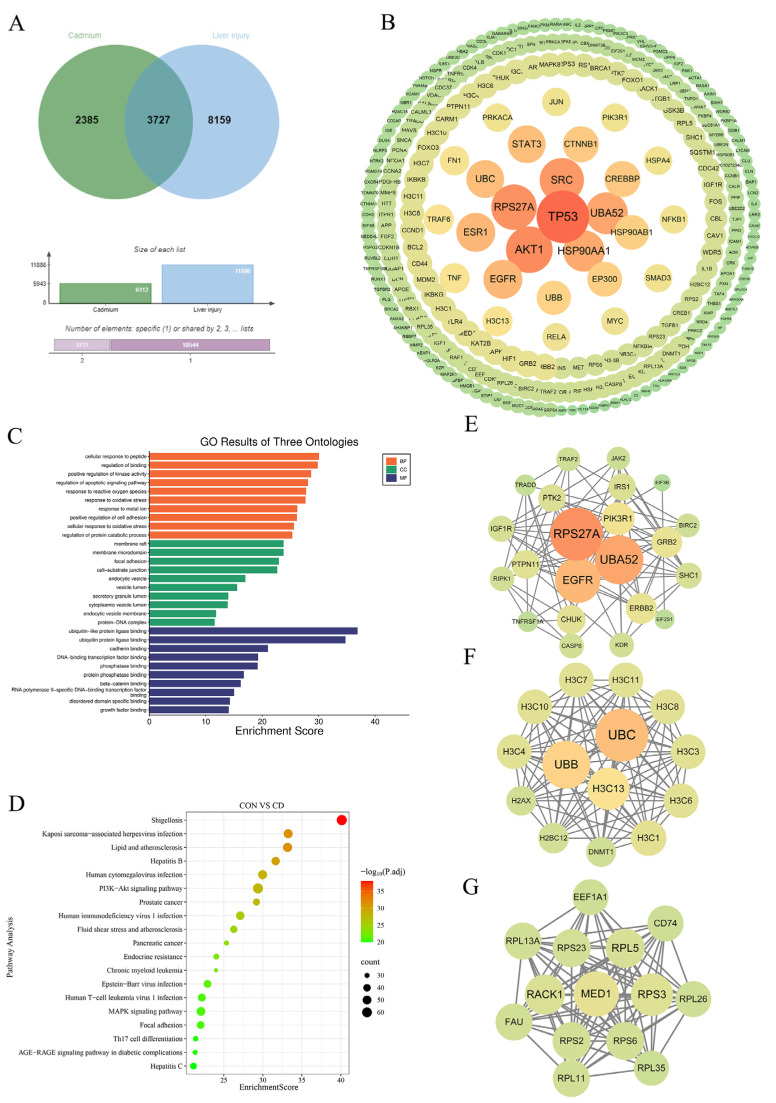
Network toxicology analysis and target prediction of Cd hepatotoxicity: (**A**) Venn diagram showing the overlap between Cd-related targets and liver injury-related targets; (**B**) PPI network of potential targets involved in Cd exposed liver injury; (**C**) GO enrichment analysis of potential targets associated with Cd exposed liver injury; (**D**) KEGG enrichment analysis of potential targets associated with Cd exposed liver injury; (**E**–**G**) PPI subnetworks of the core targets involved in Cd-exposed liver injury (top three highest-scoring subnetworks identified by Mcode).

By integrating data from multiple databases, a total of 6112 Cd-related targets and 11,886 liver injury-related targets were collected. A Venn diagram analysis was performed to identify overlapping targets, revealing 3727 shared potential targets associated with Cd-exposed liver injury (Figure 4A). To further investigate the interactions among these targets, a PPI network was constructed. Using the STRING database with a minimum required interaction score of 0.9, the core targets were selected based on the following criteria: betweenness centrality, closeness centrality, and degree > median value, resulting in a PPI network with 294 nodes (Figure 4B). These genes were identified as potential key targets involved in Cd-exposed liver toxicity and were selected for subsequent functional enrichment and network topology analyses.

### 3.5. Functional and Enrichment Analysis of Target Genes

To explore the potential mechanisms of Cd-exposed liver injury, GO and KEGG enrichment analyses were conducted on the 294 key genes identified from the PPI network. GO enrichment analysis showed that differentially expressed genes were concentrated in biological processes (BP) such as oxidative stress and metal ion response, peptide response, cell adhesion, and protein degradation metabolism, and were significantly localized to membrane rafts, focal adhesions, endocytic vesicles, and their compartments (CC). They primarily encoded ubiquitin/ubiquitin-like ligases, transcription factors, kinases/phosphatases, and growth factors, suggesting that cells respond to stress by enhancing membrane transport, protein homeostasis, and signal transduction. (Figure 4C). KEGG enrichment analysis of differentially expressed genes identified the top significantly enriched pathways, with predominant enrichment in immune dysregulation and stress-response clusters. The striking activation of multiple viral infection pathways (Kaposi’s sarcoma-associated herpesvirus, hepatitis B/C, cytomegalovirus) mirrored host transcriptional responses typically induced by viral invasion, suggesting Cd exposure creates an immunocompromised microenvironment permissive to pathogen-like signaling. Concurrently, immune-cancer axis pathways (PI3K-Akt signaling, Th17 differentiation, HTLV-1 infection) revealed Cd-exposed disruption of immune surveillance through synergistic hyperactivation of pro-inflammatory cascades and suppression of tumor-suppressor networks. Critically, the atherosclerosis–lipid cluster (lipid metabolism, fluid shear stress, AGE-RAGE) and endoplasmic reticulum stress pathway demonstrated Cd’s disruption of vascular homeostasis via oxidative stress amplification. Collectively, these perturbations depict Cd toxicity as a driver of systemic immune paralysis characterized by viral-mimetic inflammation, breakdown of antitumor immunity, and metabolic-stress synergy (Figure 4D).

### 3.6. Identification of Core Targets of Cd-Exposed Liver Injury

To further explore the core targets involved in Cd-exposed liver injury, Centrality metrics (degree, betweenness, and closeness) were computed for all nodes in Cytoscape. Median values were used as cutoffs, and genes exceeding the median for all three metrics were defined as core genes. Using the MCODE algorithm in Cytoscape (version 3.10.1; Cytoscape Consortium, San Diego, CA, USA) with default parameters except degree cutoff = 2, we identified three top-scoring modules from the PPI network (Figure 4E–G).

Modular analysis of the PPI network via MCODE clustering identified three high-scoring subnetworks central to Cd hepatotoxicity. The RTK-PI3K/MAPK signaling module, anchored by EGFR/ERBB2-PIK3R1 hubs, indicates activated proliferative and vascular remodeling pathways (Figure 4E). A chromatin remodeling cluster comprising histone H3/H2A families, DNMT1, and γH2AX reflects coordinated DNA damage response and epigenetic reprogramming (Figure 4F). The ribosomal stress module, enriched with RPS/RPL proteins and RACK1/FAU, implicates compromised translational fidelity and elevated apoptotic susceptibility (Figure 4G). These subnetworks collectively delineate Cd-exposed liver injury through integrated dysregulation of signal transduction, epigenetic control, and proteostatic maintenance.

### 3.7. Transcriptomic Analysis of Cd-Exposed Liver Tissue

**Figure 5:**
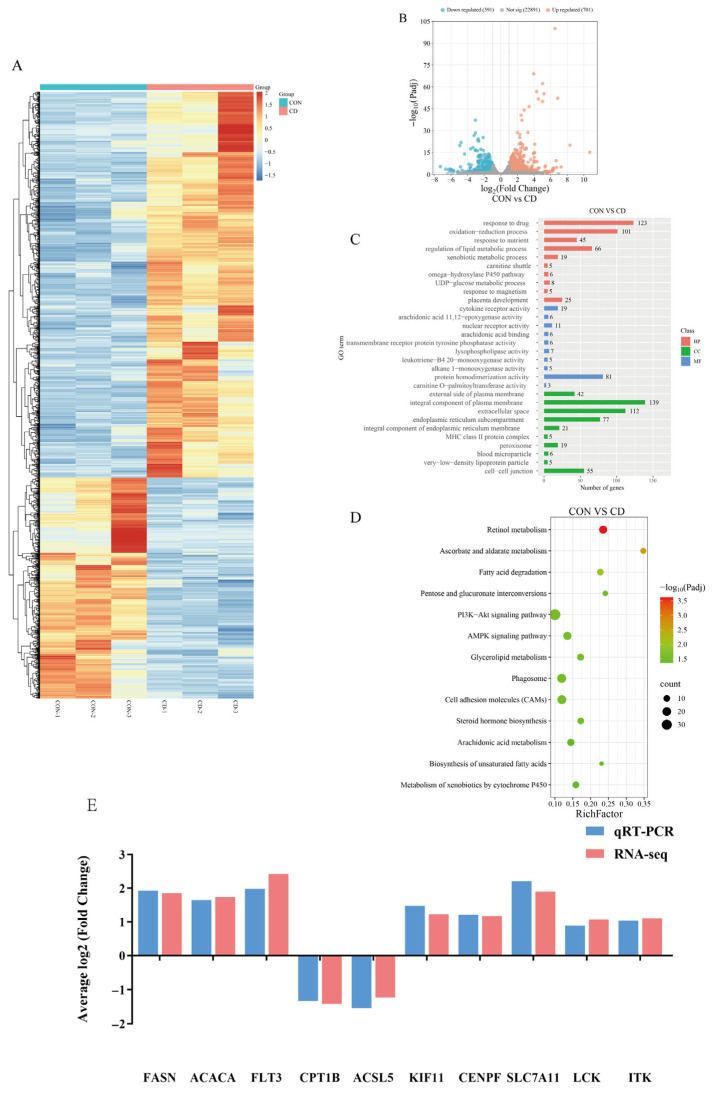
Transcriptomic analysis of Cd hepatotoxicity: (**A**) Hierarchical clustering of transcriptome profiles based on Pearson correlation coefficients. Samples are grouped by treatment: control (blue sidebar, *n* = 3) and Cd-exposed (red sidebar, *n* = 3). Dendrogram branch lengths reflect expression profile similarity. (**B**) CON vs. CD differentially expressed gene volcano plot. Among all genes, 701 were significantly upregulated (red), 391 were significantly downregulated (blue), and 22,891 did not reach significance (gray). (**C**) CON vs. CD Differential Gene GO Enrichment Analysis. The vertical axis lists biologically significant enriched biological processes, molecular functions, and cellular components; bubble size indicates the number of genes enriched in each category; color gradient corresponds to −log10 (Padj). (**D**) KEGG Pathway Enrichment of Differentially Expressed Genes Between CON and CD. RichFactor (enriched gene proportion) plotted against −log10(Padj) as a scatter plot; point size indicates the number of enriched genes. (**E**) Validation of RNA-seq data by qRT-PCR.

Transcriptome correlation analysis revealed high intra-group consistency among biological replicates but significant inter-group divergence, indicating distinct global expression patterns between conditions (Figure 5A). This clear separation suggests extensive transcriptomic reprogramming, which was subsequently confirmed by differential expression analysis.

RNA-seq identified 29,383 genes expressed in pig liver across the CON and CD groups. Differential expression analysis using Padj < 0.05 and |log2FC| > 1 yielded 1092 DEGs in CD relative to CON, including 701 upregulated and 391 downregulated genes (Figure 5B).

GO enrichment analysis (Figure 5C) of Cd-exposed liver DEGs revealed distinct stress-response patterns. The analysis revealed that differentially expressed genes were significantly enriched in key biological processes such as oxidation-reduction processes (119 genes), cytokine-mediated signaling pathways (92 genes), and regulation of lipid metabolic processes (82 genes). These results suggest that under Cd exposure conditions, cells may adapt to environmental changes by enhancing redox homeostasis regulation, activating immune signaling cascades, and remodeling lipid metabolic networks.

KEGG pathway enrichment analysis (Figure 5D) identified significantly enriched pathways among differentially expressed genes, revealing coordinated activation of interconnected functional axes central to hepatic adaptation under Cd exposure. Key enriched pathways encompassed xenobiotic metabolism through cytochrome P450, fatty acid degradation, arachidonic acid metabolism, glyceride metabolism, retinol metabolism, and ascorbate and aldarate metabolism, collectively facilitating enhanced detoxification and membrane lipid homeostasis remodeling. Simultaneously, enrichment in steroid hormone biosynthesis, phagosome activity, cell adhesion molecules, AMPK signaling, and PI3K-Akt pathways indicated reprogrammed energy sensing, inflammatory regulation, and intercellular communication. This integrated transcriptional response suggests cellular adaptation occurs via synergistic enhancement of detoxification capacity, structural lipid reconfiguration, and crosstalk between metabolic and immune signaling networks.

To validate the RNA-seq data, 10 DEGs were randomly chosen for qRT-PCR. The expression trends from qRT-PCR were consistent with the sequencing results, supporting the reliability of the transcriptomic analysis (Figure 5E).

## 4. Discussion

Cd is a pervasive environmental toxicant with well-documented hepatotoxic effects [21,22], yet the integrative mechanisms underlying its toxicity, particularly in physiologically relevant swine models, remain incompletely understood. In the present study, we employed a multi-omics approach combining histopathological, ultrastructural, biochemical, network toxicological, and transcriptomic analyses to systematically characterize Cd-exposed liver injury in weaned piglets. Our results not only reinforce established paradigms of Cd hepatotoxicity but also provide novel insights into the subcellular and transcriptomic perturbations induced by chronic Cd exposure. Compared with classical rodent models, the swine model revealed both conserved and species-specific features of Cd hepatotoxicity. Similar to rodent studies, we observed mitochondrial dysfunction, lipid droplet accumulation, and PI3K-Akt pathway suppression. However, the autophagic activation and lipid metabolic remodeling were more pronounced in pigs, consistent with their closer hepatic metabolic profile to humans. These findings reinforce the translational value of the swine model for environmental toxicology research.

Histopathological and ultrastructural analyses revealed marked hepatic architecture disorganization, vacuolar degeneration, inflammatory infiltration, and pronounced mitochondrial damage accompanied by autophagic activation. These findings are consistent with previous reports indicating Cd-exposed oxidative stress and organelle dysfunction [23,24,25]. The significant elevation in serum ALT, a specific indicator of hepatocellular injury, further corroborates the functional impairment of hepatocytes following Cd exposure.

Through integrative network toxicology, we identified 3727 overlapping targets bridging Cd exposure and liver injury, highlighting the complexity of Cd-exposed hepatotoxicity. Combining GO and KEGG results, potential target genes exhibit broad cellular protective behaviors in oxidative stress, metal ion stress, membrane transport, and protein homeostasis, while significantly enriching in viral infection, atherosclerosis, and inflammatory signaling pathways such as PI3K-Akt/MAPK. These findings collectively suggest that Cd exposure affects the membrane–endocytosis system, protein ubiquitination-phosphorylation networks, and immune–metabolic cross-regulation. Numerous studies have demonstrated that Cd exposure causes oxidative stress [5,21,26], immune and inflammatory changes [27,28], which is consistent with the results of this study. Notably, the enrichment in hepatitis B/C and PI3K-Akt pathways suggests that Cd may elicit a viral-like inflammatory response and disrupt immune–metabolic crosstalk. Furthermore, the identification of core subnetworks—RTK-PI3K/MAPK signaling, chromatin remodeling, and ribosomal stress—provides a novel framework for understanding how Cd coordinately dysregulates proliferative signaling, epigenetic stability, and translational fidelity, mechanisms previously implicated in heavy metal toxicity [29,30].

Transcriptomic profiling further validated these observations, with 1092 DEGs significantly enriched in detoxification metabolism, lipid homeostasis, immune response, and energy sensing pathways. Under Cd exposure conditions, cells remodel membrane lipid composition, regulate levels of arachidonic acid-derived inflammatory mediators, and enhance extracellular signal exchange through two main pathways: “CYP450-mediated lipid metabolism–inflammation signaling” and “PI3K-Akt/AMPK-driven energy–immune integration.” These pathways synergistically facilitate adaptive responses to environmental stimuli. These findings not only provide new candidate pathways and gene sets but also point the way for subsequent functional validation and mechanism studies. The high consistency between RNA-seq and qRT-PCR results affirms the reliability of our transcriptomic data.

While each omics layer provided valuable insights, the most significant finding emerged from their integration. The convergence of both network toxicology and transcriptomic analyses uniquely identified the PI3K-Akt signaling pathway as the central hub, a finding that would have been obscured by either approach alone. The PI3K-Akt axis is a master regulator of cellular homeostasis, critically governing processes such as cell survival, proliferation, metabolism, and autophagy [31,32], all of which were markedly perturbed in our Cd-exposed model. We postulate that Cd exposure, potentially through dysregulating upstream receptors like EGFR (a core target in our RTK-PI3K/MAPK subnetwork), impairs the precise activation dynamics of PI3K-Akt signaling [33,34]. This disruption offers a coherent molecular explanation for the key pathological phenotypes we observed by elucidating several downstream consequences. The impaired Akt signaling compromises mitochondrial integrity through the faulty regulation of pro-apoptotic factors [35], accounting for the observed swelling and cristae disintegration. Concurrently, the suppression of this pathway de-represses its tonic inhibition on autophagy, mediated through the TSC1/2 complex and mTOR [36,37], thereby triggering excessive autophagic flux marked by numerous autophagosomes. Additionally, the dysregulation of Akt disrupts metabolic homeostasis, as its role as a key activator of glycolytic enzymes and the lipogenic transcription factors SREBPs directly explains the lipid metabolic disturbances and vacuolar degeneration [38,39]. Therefore, the PI3K-Akt pathway emerges not merely as a participant but as a critical signaling nexus through which Cd disrupts hepatic energy balance, induces programmed cell death pathways, and ultimately drives hepatotoxicity [40].

**Figure 6:**
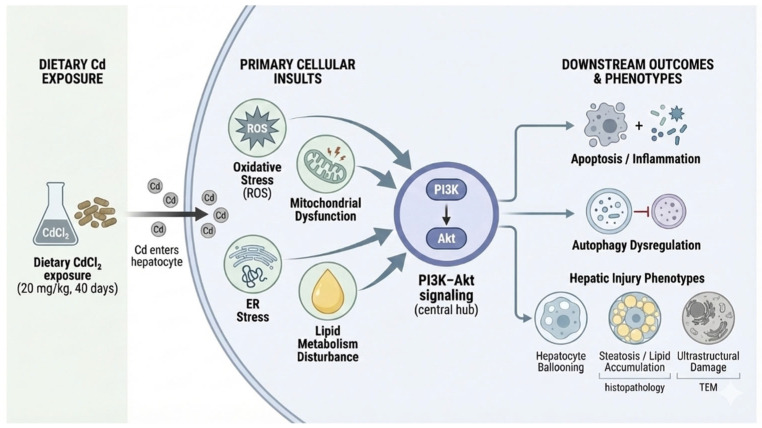
Proposed PI3K–Akt-centered mechanism of Cd-exposed hepatotoxicity in swine.

Collectively, our findings propose a model wherein Cd hepatotoxicity in swine is driven by mitochondrial dysfunction, autophagic activation, immune–inflammatory disruption, and broad metabolic reprogramming [25,37], with the PI3K-Akt pathway serving as a pivotal orchestrator of these effects (Figure 6). This study underscores the utility of the swine model in environmental toxicology, closely mirroring human hepatic pathophysiology due to high genomic homology and metabolic similarity [14]. The identified core targets (e.g., EGFR, DNMT1, RACK1) may serve as potential biomarkers for monitoring Cd exposure in agricultural and food safety contexts. These candidate markers may support risk surveillance in edible tissues (particularly liver) and thereby inform food-safety monitoring strategies in Cd-impacted production settings.

Despite the integrative insights provided by the present multi-omics approach, several limitations should be acknowledged. Notably, the internal cadmium burden, including blood and hepatic Cd concentrations, was not quantified in this study. Measurement of internal Cd levels would allow a more direct linkage between dietary exposure and tissue-specific dose, thereby strengthening toxicokinetic interpretation. However, the current work was intentionally designed as a hazard-oriented mechanistic investigation aimed at elucidating system-level molecular pathways of Cd-exposed hepatotoxicity rather than exposure–dose quantification. The consistent histopathological, biochemical, and transcriptomic alterations observed across biological replicates support the robustness of the identified signaling networks. Future studies will incorporate validated instrumental analyses (e.g., ICP-MS) and expanded cohorts to integrate internal dose assessment with functional pathway validation.

Although our multi-omics analyses consistently highlighted the PI3K–Akt pathway as a mechanistic hub, direct functional validation at the protein and signaling-activity levels was not performed. Specifically, we did not quantify key pathway components (e.g., PI3K/Akt/mTOR) or their activation status (such as phosphorylation), nor did we conduct pathway-perturbation experiments to test causality. Therefore, the proposed PI3K–Akt-centered mechanism should be interpreted as a strongly supported model derived from convergent phenotypic and transcriptomic evidence, which warrants targeted validation using protein-level assays and functional modulation approaches.

## 5. Conclusions

Cd exposure induced overt hepatocellular injury in weaned piglets, manifested by disorganized hepatic architecture with vacuolar/lipid degeneration, pronounced mitochondrial swelling with abundant autophagosomes, and marked elevation of serum ALT. Transcriptomic profiling identified 1092 DEGs enriched in xenobiotic/lipid metabolism and immune-inflammatory signaling, reflecting a coordinated metabolic–immune adaptation to Cd challenge. Critically, the convergence of network toxicology and RNA-seq uniquely pinpointed PI3K–Akt-centered signaling as the mechanistic hub linking these transcriptional programs to the observed toxicological phenotypes, integrating mitochondrial dysfunction, dysregulated autophagy, and metabolic imbalance into a unified injury framework. This multi-layer evidence supports PI3K–Akt-related targets (EGFR, DNMT1, and RACK1) and downstream metabolic markers as candidate biomarkers for Cd-associated hepatic hazard surveillance in swine production chains. While the robust pathological and transcriptional phenotypes validate this exposure model, future studies incorporating internal Cd burden measurement and functional validation in larger cohorts are essential for translational application and risk assessment.

## Data Availability

The RNA-seq data generated in this study have been deposited in the China National Center for Bioinformation (CNCB) under accession number PRJCA055268. Other data supporting the findings are available from the corresponding author upon reasonable request.

## Appendix A

**Table A1:** Primer information.

Genes	Primer Sequences (5′-3′)	Accession Number
FASN	F:CCCGAATCTGCACTACCACA R:AGCCGAAGGAGTTTATGCCC	NM_001099930.1
ACACA	F:ATTGGGGCTTACCTTGTCCG R:AGCTGGCTAGTGGAGGTGT	NM_001114269.1
FLT3	F:GAATGGGTGTTCTGCGATGC R:AATAACTGTGGCGGTGTGGT	XM_021065521.1
CPT1B	F:TGGGGCTGGTCAATCACATC R:TGCCAGCATAGGGTTTGGTT	NM_001007191.1
ACSL5	F:ATGGGCCTTGCTTGGGATAC R:TTCGTCTGGTGATGGCTTGT	NM_001195321.1
KIF11	F:TCTAGTGGTGGGGTTCCCTT R:TTGTTCTGGGCTCGTAGAGG	XM_003133148.4
CENPF	F:GAACACGCGCAACATCCTAC R:TCACGAGGCTGTTTTCCCTC	XM_003130395.6
SLC7A11	F:AATGGTGGTGTGTTTGCTGTCTC R:GAGGAGTGTGTTTGCGGATGTG	XM_021101587.1
LCK	F:CAGCACCAGAAGCCATCAACTAC R:GATGCGACCGTGAGTGACAATC	NM_001143713.1
ITK	F:ACGCTGGTCATTGCCTTGTA R:CACGTATCCTTCATGCCCGT	XM_021076697.1
β-actin	F:TCGGAGTGAACGGATTTGGC R:GACAAGCTTCCCGTTCTCC	XM_021086047.1
